# Retraction: Liu, W., et al. Ailanthone Induces Cell Cycle Arrest and Apoptosis in Melanoma B16 and A375 Cells. *Biomolecules* 2019, *9*, 275

**DOI:** 10.3390/biom10040627

**Published:** 2020-04-17

**Authors:** Biomolecules Editorial Office

**Affiliations:** MDPI, St. Alban-Anlage 66, 4052 Basel, Switzerland; biomolecules@mdpi.com

The Editorial Office and the authors have taken the decision to retract the published article [1]. The Western blot data shown in Figures 6a and 6c are unacceptably similar, in particular the backgrounds show identical features. The published paper [1] will therefore be marked as retracted.

MDPI is a member of the Committee on Publication Ethics and takes seriously its responsibility to publish only high-quality research. We regret that this issue was not identified earlier and apologize to readers of the journal.
